# From Two-Phase to Three-Phase: The New Electrochemical Interface by Oxide Electrocatalysts

**DOI:** 10.1007/s40820-017-0161-5

**Published:** 2017-10-23

**Authors:** Zhichuan J. Xu

**Affiliations:** 1 School of Materials Science and Engineering, Nanyang Technological University, Singapore, 639798 Singapore; 2 Solar Fuels Lab, Nanyang Technological University, Singapore, 639798 Singapore; 3 Energy Research Institute@NTU (ERI@N), Nanyang Technological University, Singapore, 639798 Singapore

**Keywords:** Electrochemistry, Interface, Oxides, Carbon, Electrocatalyst

## Abstract

Electrochemical reactions typically occur at the interface between a solid electrode and a liquid electrolyte. The charge exchange behaviour between these two phases determines the kinetics of electrochemical reactions. In the past few years, significant advances have been made in the development of metal oxide electrocatalysts for fuel cell and electrolyser reactions. However, considerable gaps remain in the fundamental understanding of the charge transfer pathways and the interaction between the metal oxides and the conducting substrate on which they are located. In particular, the electrochemical interfaces of metal oxides are significantly different from the traditional (metal) ones, where only a conductive solid electrode and a liquid electrolyte are considered. Oxides are insulating and have to be combined with carbon as a conductive mediator. This electrode configuration results in a three-phase electrochemical interface, consisting of the insulating oxide, the conductive carbon, and the liquid electrolyte. To date, the mechanistic insights into this kind of non-traditional electrochemical interface remain unclear. Consequently conventional electrochemistry concepts, established on classical electrode materials and their two-phase interfaces, are facing challenges when employed for explaining these new electrode materials.

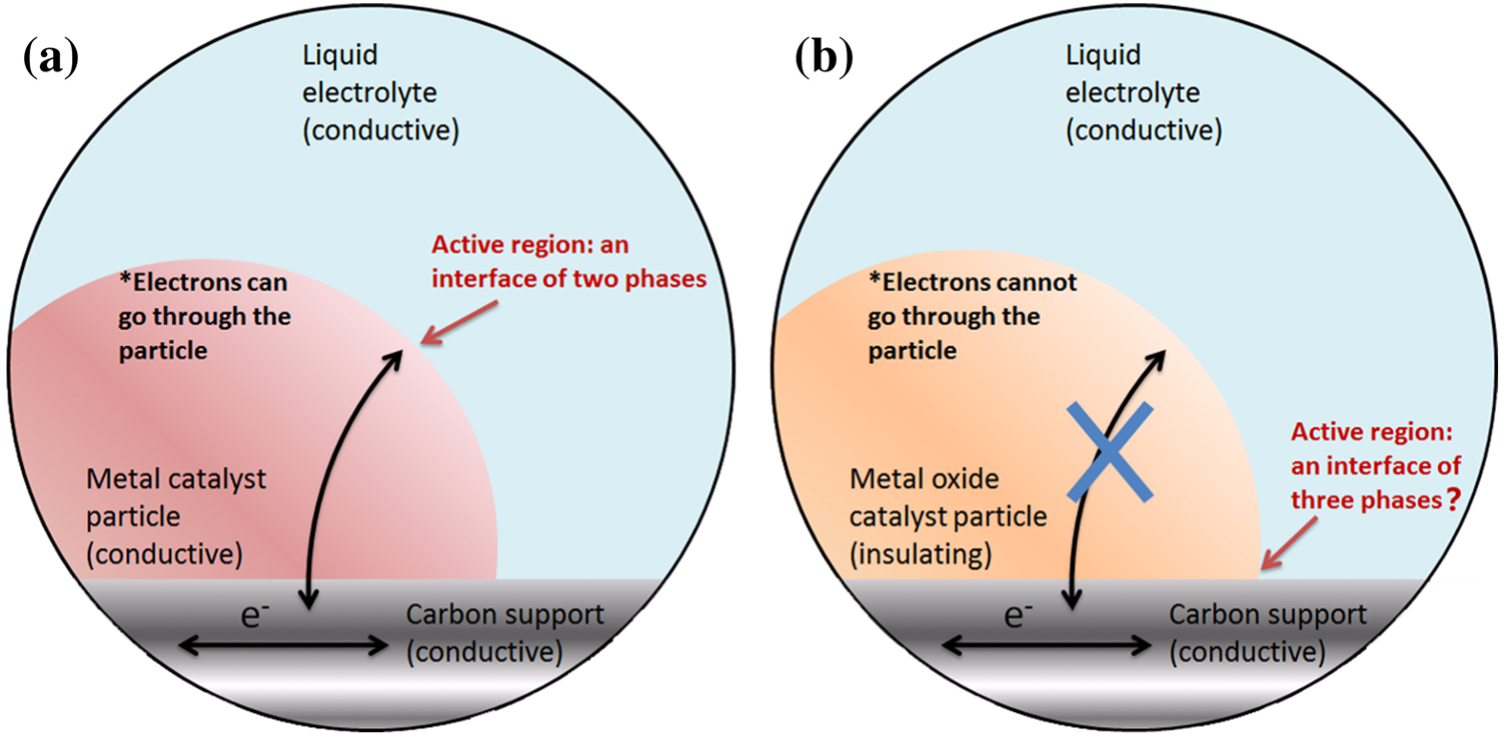

The central phenomenon of an electrochemical process is the charge exchange between an electronically conductive phase and an ionically conductive phase. The boundary of these two phases is the electrochemical interface. Usually such an interface is more than a flat surface. The “surface” can be very rough and even porous. The regions in both phases near the interface, in depth from a few Å to a few hundred nanometres, have to be considered as well as the part of the interface. Thus, it should be noted that the regions are of three-dimensional nature, although they are called “the interface” [1]. A classical electrochemical interface consists of a solid, conductive electrode, and an electrolyte. The electrolyte can be either liquid or solid. Conventionally, a typical electrochemical interface is considered to consist of two phases: the solid electrode and the liquid electrolyte. This is particularly true for conducting bulk electrodes, where no additional component is involved. For non-bulk electrodes, like particulate catalysts, a catalyst support, e.g. carbon black, has to be used to ensure high dispersion of catalyst particles for the maximum utilisation of the catalyst surface [2]. The most studied catalysts are metals, e.g. Pt, Pd, PtNi, PtCo. [3–5]. These metallic catalysts are intrinsically conductive, and the electrochemical interfaces are still considered as a two-phase interface, where only the metallic catalyst and the liquid electrolyte are considered. The influence of the additional support phase (the carbon support) to the interface is usually ignored, and its major function is for dispersing catalyst particles. This is not an unreasonable perception, since electrons can flow through the metallic catalyst (Fig. 1a).

**Fig. 1:**
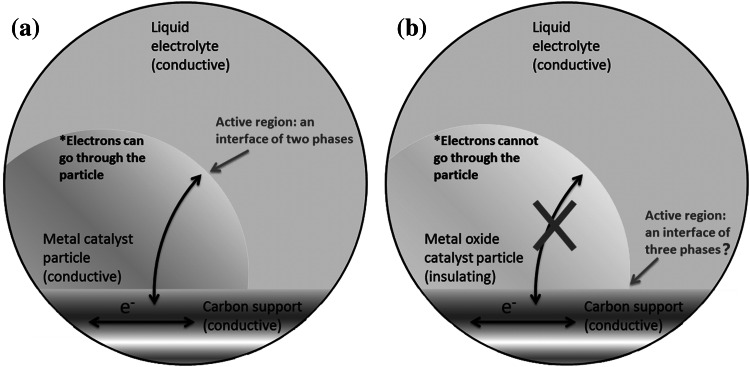
Schematic display of the difference between the electrochemical interfaces of **a** a carbon-supported metal and **b** a metal oxide

However, this view needs to be revised when the catalyst particles are no longer metallic. In recent years, there has been a worldwide effort to explore metal oxides as electrocatalysts for reactions of small molecules, like ORR (oxygen reduction reaction) and OER (oxygen evolution reaction), which are key reactions for determining the efficiency of devices like fuel cells and water electrolysers, utilising renewable energy sources. Although most metal oxides are poor in electrical conductivity, exciting progress has been made and many metal oxides have been found very active for those reactions [6–9]. Some of them even show an activity comparable to noble metals [10, 11]. For example, Mn- and Co-spinel [9] and perovskite [6, 7] oxides have shown promising activity for ORR and OER [12–14]. Because of their insulating nature, these oxide catalyst particles (either nano-sized or micro-sized) have to be mixed with carbon for application as an electrode. Some research also demonstrates that employing graphene as a carbon support can further improve the activity of metal oxide catalysts although it is unclear how this improvement occurs. Considering the physics of this, the claim that the major contribution stems from the interface metal oxide/liquid electrolyte may be at best questionable. Since these oxides are not conductive, a passage of electrons through the oxide is not a thermodynamically favourable option (Fig. 1b). The electrochemically active region is more likely to be located where the reactants can reach the catalyst as well as the electrons. Thus, the electrochemical three-phase boundary (the metal oxide, the carbon support, and the electrolyte) cannot be ignored and it is highly likely to be a critical site of electrochemical reactivity.

At such a three-phase interface, the carbon should have played more important role in the whole catalytic process besides the functions of electron transport and particle support. To date, this role has not been discovered. Most historical research has focused on new oxide catalyst development and performance improvement over the existing oxide catalysts [10–12]. There is limited effort on the fundamental investigation of three-phase electrochemical interface. In particular, graphene is the most interesting carbon support, which has been widely used to replace the traditional carbon support, like Vulcan carbon and acetylene black. There are literally tons of research papers reporting the significant catalytic performance enhancement of graphene supported versus carbon black or carbon nanotube supported the same catalysts. Interestingly, the enhancement most often is explained by a synergistic effect between graphene and catalyst particles. However, what the meaning of this synergistic effect remains unclear. Some researchers ascribed it to the higher conductivity of graphene, but this is doubtful. In principle, although graphene has a high conductivity, the “graphene” used as catalyst support is actually reduced graphene oxide, which contains *sp* ^3^ carbons and its conductivity is actually lower than that of graphite (2400 ± 200 S m^−1^ for reduced graphene oxide and 2500 ± 20 S m^−1^ for graphite) [13]. When used in powder form and as catalyst support, graphene does not show a significant difference in conductivity as compared to other carbons, like acetylene black. Thus, the conductivity should not be the origin for that widely reported synergistic effect.

Recently, we compared graphene with acetylene black and carbon nanotubes in terms of their influence on the redox ability of Mn cations in supported MnO_2_ [14]. By subtracting the capacitance contribution from the carbon support, we found that graphene may enhance the pseudo-capacitance of MnO_2_, indicating an improved redox ability of MnO_2_ by graphene. However, at high scan rates, both acetylene black and carbon nanotubes are better than graphene for MnO_2_ to deliver its capacitance. This observation should not be ascribed to the electron conductivity difference of these carbons, but more likely caused by the differences in their morphology and surface chemistry.

To conclude, from metallic catalysts to oxides, the electrochemical interface has evolved from the classical two-phase interface to a three-phase one. It is necessary to put more effort on investigating these non-traditional electrochemical interfaces in oxide electrocatalysts. The attention may be given to the surface chemistry of various carbon supports as well as the interaction between oxides and carbons. Probably by well-designed model experiments, plus advanced characterisation tools, such as in situ Raman spectroscopy, scanning electrochemical microscopy, X-ray absorption fine structure, the community will be able to eventually clarify the vagueness in explaining the performance enhancement of some hybrid and novel oxide/carbon electrocatalysts.
